# Genetic variation and population structure in a threatened species, the Utah prairie dog *Cynomys parvidens*: the use of genetic data to inform conservation actions

**DOI:** 10.1002/ece3.1874

**Published:** 2016-01-08

**Authors:** Nathanael L. Brown, Mary M. Peacock, Mark E. Ritchie

**Affiliations:** ^1^ Department of BiologySyracuse University107 College Place, LSCSyracuseNew York13224 Mark Ritchie; ^2^ Department of Biology MS314University of Nevada Reno1664 North Virginia StreetReno89557Nevada; ^3^ Utah Field OfficeUnited States Fish and Wildlife Service1789 N. Wedgewood LaneCedar CityUtah84721

**Keywords:** Bayesian genotype clustering analysis, genetic diversity, genetic structure, threatened, Utah prairie dog

## Abstract

The Utah prairie dog (*Cynomys parvidens*), listed as threatened under the United States Endangered Species Act, was the subject of an extensive eradication program throughout its range during the 20th century. Eradication campaigns, habitat destruction/fragmentation/conversion, and epizootic outbreaks (e.g., sylvatic plague) have reduced prairie dog numbers from an estimated 95,000 individuals in the 1920s to approximately 14,000 (estimated adult spring count) today. As a result of these anthropogenic actions, the species is now found in small isolated sets of subpopulations. We characterized the levels of genetic diversity and population genetic structure using 10 neutral nuclear microsatellite loci for twelve populations (native and transplanted) representative of the three management designated “recovery units,” found in three distinct biogeographic regions, sampled across the species' range. The results indicate (1) low levels of genetic diversity within colonies (*H*_e_ = 0.109–0.357; *H*_o_ = 0.106‐ 0.313), (2) high levels of genetic differentiation among colonies (global *F*_ST_ = 0.296), (3) very small genetic effective population sizes, and (4) evidence of genetic bottlenecks. The genetic data reveal additional subdivision such that colonies within recovery units do not form single genotype clusters consistent with recovery unit boundaries. Genotype cluster membership support historical gene flow among colonies in the easternmost West Desert Recovery Unit with the westernmost Pausaugunt colonies and among the eastern Pausaugunt colonies and the Awapa Recovery unit to the north. In order to maintain the long‐term viability of the species, there needs to be an increased focus on maintaining suitable habitat between groups of existing populations that can act as connective corridors. The location of future translocation sites should be located in areas that will maximize connectivity, leading to maintenance of genetic variation and evolutionary potential.

## Introduction

Habitat loss and range reduction are among the most serious threats to species persistence (Dunham et al. 1997; Collier et al. 2010; Nuria et al. 2012; Rogers and Peacock 2012; Agnarsson et al. 2013; Gottelli et al. 2013; Venturas et al. 2013). It is well appreciated that small isolated demographic units are particularly vulnerable to random genetic drift and concomitant loss of genetic variation (Frankham 2005). Under scenarios of global climate change, small isolated populations are likely to experience increased extinction probabilities due to reduced evolutionary potential (Peacock and Dochtermann 2012), which is dependent upon heritable genetic variation in adaptive traits (Naish and Hard 2008; Robinson et al. 2008; Naish et al. 2013; Olson et al. 2013). The conservation genetics literature is replete with examples of habitat loss, fragmentation, and reductions in genetic variation from a wide variety of taxa including once widely dispersed species [e.g., mountain lion (*Puma concolor*; Ernest et al. 2003); white rhinoceros (*Ceratotherium simum*; Nielsen et al. 2008); Siberian flying squirrel (*Pteromys volans*; Lampila et al. 2009); European ground squirrels (*Spermophilus citellus*; Ben Slimen et al. 2012; ] as well as narrowly distributed endemics [e.g., mouse lemur species (*Microcebus* spp.; Olivieri et al. 2008); Devils Hole pupfish (*Cyprinodon diabolis*; Martin et al. 2012); and bluemask darter (*Etheostoma akatulo*; Robinson et al. 2013)]. Conservation strategies for species with reduced genetic variation are generally aimed at maximizing the maintenance of the remaining genetic variation (Peacock et al. 2010; Ernst et al. 2013; Mondol et al. 2013; Schueler et al. 2013).

For species with distinct evolutionarily significant units (ESUs) designated, the challenge is not only to maintain the coadapted gene complexes which are thought to define each unit, but also to maximize the species‐level genetic diversity in the face of declining population numbers. ESUs have been defined in a number of ways including Moritz's (1994) reciprocal monophyly for mtDNA alleles Fraser and Bernatchez (2001). However, in practice, genetically differentiated populations found in differing habitats are often grouped into distinct ESUs whether adaptive differences have been demonstrated or not (Peacock and Dochtermann 2012). Fraser and Bernatchez (2001) outline an *adaptive evolutionary conservation* approach which aims to provide a more unified concept that includes both genetic and ecological considerations and a more flexible species‐specific approach. At present, the International Union for Conservation of Nature (IUCN) guidelines for within ESUs, however they are defined, management practices include reestablishing extirpated populations with individuals from the closest intact populations (Hoogland et al. 2011; May et al. 2011; Rosell et al. 2012). Only in cases of extensive population loss within an ESU would recolonization of habitat with individuals from outside the ESU be considered (Halley 2011). However, in practice the mixing of gene pools from separate ESUs is not typically undertaken (Peacock et al. 2010; Paplinska et al. 2011). However, highly depauperate gene pools may require genetic rescue such as in the extreme case of the Florida panther (*Puma concolor coryi*), where fixation of deleterious alleles and inbreeding depression warranted interbreeding between subspecies (*P. c. stanleyana x P.c. coryi*; Pimm et al. 2006; Johnson et al. 2010). Johnson et al. (2010) further emphasize that without additional habitat the genetic rescue of the Florida panther will not be sufficient to conserve this subspecies. For highly endangered species such as the South Island robin (*Petroica australis*), where there were no outbred populations to draw upon, reciprocal translocations between inbred populations were used to genetically rescue this species (Heber et al. 2013). As wild populations face new challenges associated with global climate change, strategies for the conservation of genetic resources must include consideration of evolutionary potential in addition to maintenance of ESU‐based coadapted gene complexes, whose “fitness benefits” may be tied to environments that are rapidly changing.

In western North America, many ground‐dwelling sciurids including all five of the prairie dog species (Gunnison's prairie dog, *Cynomys gunnisoni*; white‐tailed prairie dog, *C. leucurus*; black‐tailed prairie dog, *C. ludovianus*; Mexican prairie dog, *C. mexicanus* and Utah prairie dog, *C. parvidens*) were the target of extensive poisoning regimes in the 19th and 20th centuries. These species were thought to be competitors for forage with open range livestock, and their extensive burrow systems were deemed an injury risk for cattle. Ironically, recent research shows that cattle preferentially graze along prairie dog colony edges and use their colony centers for resting, similar to the mutualistic relationship prairie dogs once had with the American bison (Sierra–Corona et al. 2015).

As a result of extermination campaigns, many of these once widespread species are either candidates for listing or are now federal or state listed as threatened or endangered: Franklin's ground squirrel *Spermophilus franklinii*, Mexican prairie dog; Mohave ground squirrel *Spermophilus mohavensis*, Northern Idaho ground squirrel *Spermophilus brunneus brunneus*, Townsend's ground squirrel *Urocitellus townsendii townsendii*, Utah prairie dog, and Washington ground squirrel *Urocitellus washingtoni* (http://www.fws.gov/endangered/).

**Figure 1:**
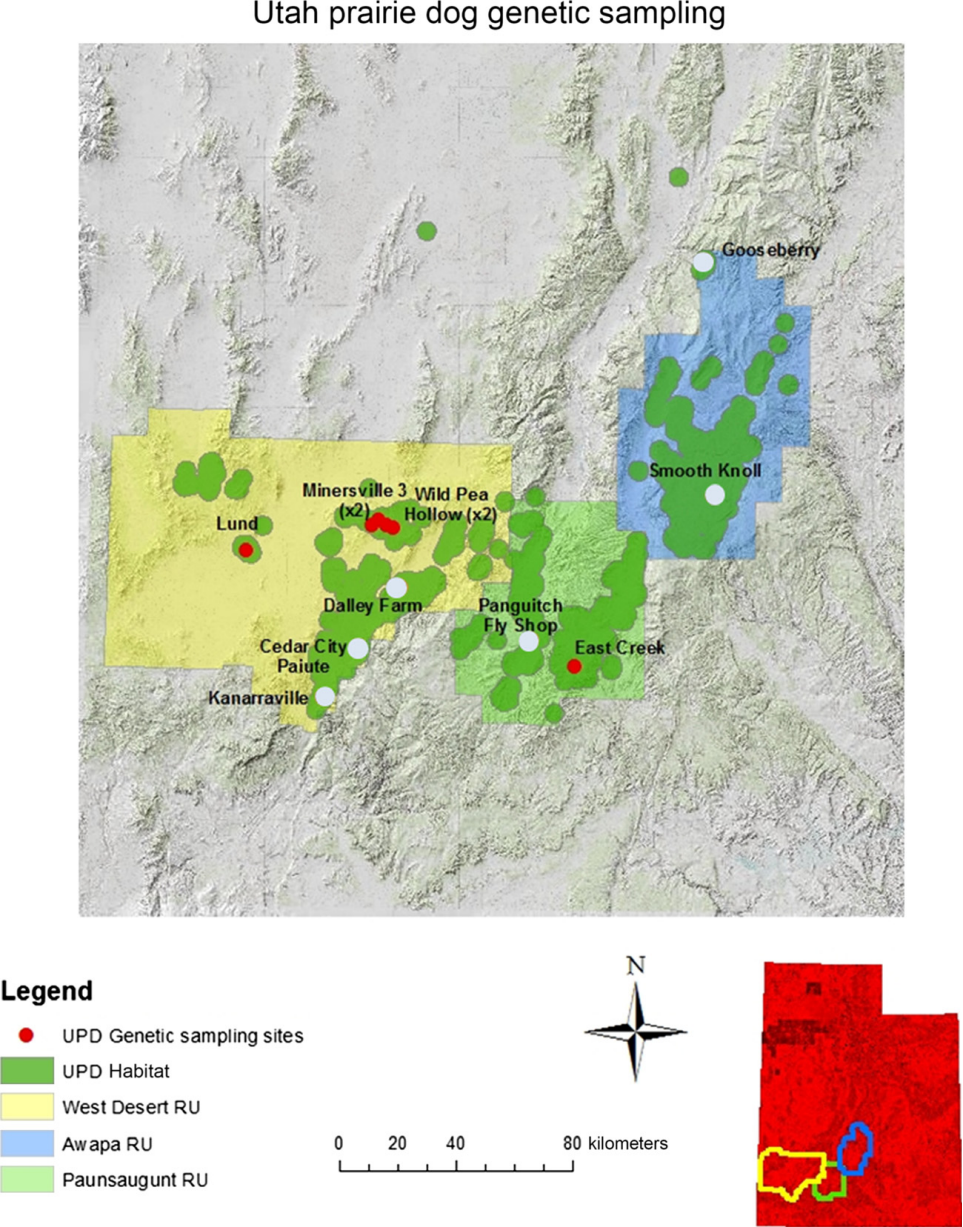
Geographic distribution of the Utah prairie dog in southwest Utah with three recovery units indicated; Awapa Plateau, Paunsaugunt, and West Desert. All native colonies are indicated with a white dot and transplanted colonies with a red dot.

Here we focus on declines in the Utah prairie dog and examine genetic variability in the context of evolutionary potential for this species under current management practices. The Utah prairie dog underwent significant declines due to a particularly draconian pest control regime during the 20th century. These efforts together with declines associated with the sylvatic plague (*Yersinia pestis*) introduced into North America in 1899 dramatically reduced the number of individuals. In the 1920s, the number of Utah prairie dog was estimated at ~95,000 individuals, but by the 1960s the population had been reduced substantially and was estimated at <3300 individuals occupying 37 colonies in 1972 (Collier and Spillett 1973). The species currently exists in eight counties in southwest Utah occupying three “recovery units” delineated along three separate biogeographic regions (see Fig. 1).

The species was listed as endangered on June 4, 1973 under the United States Endangered Species Act (ESA). An increase in individuals on private land allowed the species to be reclassified as threatened on May 29, 1984 [United States Fish and Wildlife Service (USFWS) 1991]. Recovery activities have been underway since 1972 where the principle strategy has been the translocation of prairie dogs from private and agricultural land to public land where they can be protected and managed more effectively. Prairie dogs play an important role in the ecosystem processes and ecosystem function of the habitats they occupy and because they alter nutrient cycling regimes, foraging behavior of domestic and native ungulates, and local plant species composition, they are also considered a “keystone species” (Whicker and Detling 1988; Ceballos et al. 1999; Miller et al. 2000; Magle and Crooks 2008). Whether they are a true “keystone” species (Kotliar 2000; Miller et al. 2000) or not, Utah prairie dogs serve as an “umbrella” species for the conservation of a robust sage‐steppe system in Southwestern Utah (United States Fish and Wildlife Service 2012). Additionally, a suite of species utilize Utah prairie dog burrows or depend upon prairie dogs as prey, including the burrowing owl (*Athene cuniculaira*), ground squirrels (*Spermophilus spp*.), and mustelids (*Mustelidae,* badgers and weasels) (United States Fish and Wildlife Service 2012). As a result of the elimination campaign, the translocation strategy, and continued habitat modifications (destruction, fragmentation and land use conversion), Utah prairie dogs are now found in only a few sets of local populations. These populations (colonies) are found in spatially structured habitat patches, which may be completely isolated, exhibit source–sink dynamics, or function as metapopulations.

The objectives of this study were to (1) determine the level of genetic variation retained within this species at neutral nuclear microsatellite loci, (2) compare the levels of genetic variation within native colonies to colonies comprised of transplanted individuals, and (3) assess population genetic structure within and among designated “recovery units.” We assessed the levels of genetic diversity at 10 dinucleotide nuclear microsatellite loci. We used a Bayesian genotype clustering approach to define distinct genetic groups. We also tested for genetic bottlenecks, estimated effective population sizes (*N*_e_) within colonies and recovery units, and characterized genetic divergence among colonies and recovery units.

## Materials and Methods

### Study area

Twelve colonies were sampled throughout the extant range of the Utah prairie dog. Six colonies were remnant, native populations, while six colonies were the product of transplant efforts by the Utah Division of Wildlife Resources (Table 1). The majority of translocations took place from the 1970s to the 1990s. From 1972 through 1991, 15,937 prairie dogs were translocated to 38 different sites on public lands. Of those 38 translocation sites, 17 (45%) had prairie dogs present in 1992, with an average of 60 dogs counted at each site – with a range of 7–216 animals (McDonald 1993; United States Fish and Wildlife Service 2012). Through 2008, 23,359 Utah prairie dogs were translocated from private to public lands (McDonald 1993; Bonzo and Day 2003; Brown pers. comm. 2009). As of 2009, 14 of 20 translocation sites in the West Desert Recovery Unit, six of eight colonies in the Paunsaugunt Recovery Unit and four of eight colonies in the Awapa Plateau Recovery Unit were occupied (Brown pers. comm. 2009).

**Table 1:** All sampling locations, recovery unit, native or transplanted status, and population of origin, number (*N*) of adults and juveniles sampled and mean annual colony size (1989–2005)

Recovery unit	Population/colony	Transplant origin	*N* adults/juveniles	Mean annual size (individual counts) ± SD
Awapa Plateau	Smooth Knolls (SN – *native*)	_	5/4	3.63 ± 4.06
Awapa Plateau	Gooseberry (GB – *native*)	_	5/13	31.85 ± 41.13
Paunsaugunt	Panguitch Fly Shop (PF – *native*)	_	8/23	5 ± 5.76
Paunsaugunt	East Creek (EC – *transplant*)	Cedar City Paiute	11/16	22.79 ± 21.29
West Desert	Kanarraville (KV ‐ *native*)	_	9/17	107 ± 128.25
West Desert	Cedar City Paiute (CCP – *native*)	_	6/22	24.67 ± 14.47
West Desert	Dalley Farm (DF – *native*)	_	4/24	192.8 ± 94.14
West Desert	Wild Pea Hollow 1 (WP1 – *natural colonization*)	Cedar City Paiute^a^	6/20	52.1 ± 47.42
West Desert	Wild Pea Hollow 2 (WP2 – *natural colonization*)	Cedar City Paiute^a^	7/19	9 ± 3.29
West Desert	Minersville31 (M31 *– transplant*)	Cedar City Paiute	7/24	14.33 ± 10.15
West Desert	Minersville32 (M32 – *transplan*t)	Cedar City Paiute	2/27	204.3 ± 122.8
West Desert	Lund (LUND – *transplant*)	Cedar City Paiute	14/14	21.63 ± 14.6

a Wild Pea Hollow prairie dogs are thought to have colonized naturally from geographically proximate colonies.

Colonies were sampled to include sites from many habitat types, land ownership classes, and land use practices. In practice, site selection was limited by land ownership (private or public), access to colonies and by the need for colonies to be sufficient size to trap a target goal of 25–30 individuals per site. Sites were selected to incorporate colonies from each of the three recovery units as well as urban and nonurban sites.

The three recognized recovery units are separated by what may represent biogeographic barriers (mountains, forests, and red rock canyons) (Fig. 1). Such landscape features may serve to limit dispersal and thus gene flow among populations. In addition, each recovery unit features a different elevational gradient which may shape environmental heterogeneity among recovery units, for example, precipitation and temperature regimes. The West Desert recovery unit encompasses habitat from 1500 to 1800 m, the Paunsagunt Plateau from 1800 to 2400 m, and the Awapa Plateau 2100 to 3000 m. The West Desert recovery unit is separated from the Paunsaugunt recovery unit by Cedar Mountain, the Hurricane Cliffs, and the forested Markagunt Plateau. The Paunsaugunt recovery unit is separated from the Awapa Plateau by the Escalante Mountains, the East Fork Sevier River Gorge, and Parker Mountain.

Designation of the three recovery units was based on the concept of representation, redundancy, and resiliency. Representation refers to spatially capturing the ecological elements of the species across its entire range to ensure the species' adaptive capabilities are conserved. The three recovery units encompass current and historical population and habitat distributions including sufficient habitat coverage in order to provide connectivity among colonies.

### Sample collection

A scientific collection permit for trapping and tissue collection was obtained from the USFWS (#TE074705) and an Institutional Animal Care and Use (IACUC) protocol approved by Syracuse University IACUC committee. We used Tomahawk live traps baited with sweet feed and/or peanut butter/apple mix to capture individual prairie dogs. Traps were set at active burrow entrances and included multiple coteries within a colony. We calculated relatedness among adults and juveniles per colony to assess sampling of family groups (see below). Ear tissue (5–10 mg) was collected from trapped animals using an 8 mm ear notcher. Tissue samples were frozen at –20 and –80°C upon return to the laboratory from the field. Reproductive status, weight, and age class were recorded at time of capture. Spatial data were collected using a GPS unit (Garmin Etrex Venture), and waypoints were collected around the trapping area in each colony sampled. Utah prairie dogs were trapped 6/27 – 8/12 in 2003 and 6/25 – 8/4 in 2004.

### Microsatellite markers

No microsatellite markers were available for this species; therefore, we used microsatellite loci developed for other sciurids which successfully amplified in the Utah prairie dog (GS12, GS14, GS17, GS20, and GS22, GS26, Columbian ground squirrels (*Spermophilus columbians*), Stevens et al. 1997; IGS1, IGS6, the Northern Idaho ground squirrel (*Spermophilus brunneus brunneus*), May et al. 1997; EAM35, EAM163, yellow‐pine chipmunk (*Tamias amoenus*), Schulte‐Hostedde et al. 2000; Table 2). We used Micro‐Checker (version 2.2.3 Van Oosterhout et al. 2004) to test for allelic dropout and null alleles. Any locus which showed systematic patterns of deviation from HWE or had evidence of null alleles across all sampling locations was removed from the analysis.

**Table 2:** Microsatellite loci, primer sequences, repeat motif, number of alleles observed in this study, annealing temperature and literature source for all loci used in study

Locus	Primer 5′–3′	Repeat	No. of alleles	Annealing Temp (°C)	Source
GS12	F: CCAAGAGAGGCAGTCGTCCAG	(TG)_21_	2	60	Stevens et al. (1997)
	R: TCAGAGCAGAGCACTTACAGA				
GS14	F: CAGGTGGGTCCATAGTGTTAC	(TG)_30_	3	56	Stevens et al. (1997)
	R: TTGTGCCTCAGCATCTCTTTC				
GS17	F: CAATTCGTGGTGGTTATATC	(TG)_16_	3	56	Stevens et al. (1997)
	R: CTGTCAACCTATATGAACACA				
GS20	F: TCCAGAGTTTTTCAGACACA	(TG)_15_	2	66	Stevens et al. (1997)
	R: GCCCAGCCATCACCCTCACC				
GS22	F: TCCCAGAGAACAACATCAACAG	(TG)_18_	3	64	Stevens et al. (1997)
	R: TCCGCACAGGTCTTGGACTT				
GS26	F: CCCAGGGACCACATAGGAGGTA	(TG)_17_	4	60	Stevens et al. (1997)
	R: AGGACTGGGGTTGTAGGTGAGT				
IGS1	F: ATAACAGCACCCTGCTCCAC	(CA)_20_	5	68	May et al. (1997)
	R: AATCCATCCTCTACCTGTAATGC				
IGS6	F: GGGCATTAATTCCAGGACTT	(CA)_28_	4	60	May et al. (1997)
	R: GGGCTGGAATTAAAGGTATCA				
IGS1	F: ATAACAGCACCCTGCTCCAC	(CA)_20_	5	68	May et al. (1997)
	R: AATCCATCCTCTACCTGTAATGC				
EAM35	F: ATCCGTTTAGTCTGTTATGTCTCA	(TG)_12_	2	59	Schulte‐Hostedde et al. (2000)
	R: TTTAATCTAAAGGACAACAATTGC				
EAM163	F: GCCCATCAATAGTTGAATGGATA	(TC)_6_G(TC)_5_G(TC)_9_(AC)_20_	3	59	Schulte‐Hostedde et al. (2000)
	R: CCTGGAAATGCCATAATTTTATTC				

### DNA isolation, PCR conditions, and allele scoring

DNA was isolated from ear tissue using Qiagen DNeasy Tissue kits (QIAGEN INC., Valencia, California) and quantified using a Labsystems Fluoroskan Ascent fluorometer. PCR amplification was performed using a MBS Satellite 0.2G thermocycler (Thermo Electron Corporation). PCR amplification for GS12, GS26, IGS6, EAM35, and EAM163 was carried out in 15 *μ*L reaction volumes containing 15–20 ng of DNA, 2 *μ* m of each primer, and 2× Qiagen multiplex mix (containing Multiplex buffer, HotStarTaq DNA polymerase, and a 0.3 mm dNTP mix). GS12, GS26, IGS6, EAM35, and EAM163 were amplified with 33 cycles of 94°C for 30 sec, followed by annealing temperatures of 60°C (GS12, GS26, IGS6) and 59°C (EAM35, EAM163) for 90 sec, then a 30 sec extension at 72°C, followed by a 30‐min extension at 62°C. Betaine (3 *μ*L) and BSA (0.6 *μ*L) were added to the IGS1 reaction mix to decrease stutter. PCR amplification for IGS1 was carried out in 15 *μ*L volumes containing 15–20 ng of DNA, one unit of 50× Titanium Taq DNA polymerase (CLONTECH, Palo Alto, CA), 0.2 *μ* m of forward and reverse primer, 10× titanium Taq buffer, 0.3 mm dNTPs, Betaine (3 *μ*L), and BSA (0.6 *μ*L) and was brought to final volume with ddH_2_0. IGS1 was amplified using two‐step PCR with 33 cycles of 95°C for 30 sec, an annealing temperature of 68°C for 90 sec, then an extension at 72°C for 30 sec, followed by a 30‐min extension at 72°C. PCR amplification for GS14, GS17, GS20, and GS22 was carried out in 15 *μ*L volumes containing 15–20 ng of DNA, one unit of 50× Titanium Taq DNA polymerase (CLONTECH, Palo Alto, CA), 0.2 *μ* m forward and reverse primer, 10× titanium Taq buffer, and 0.3 mm dNTPs and brought to final volume with ddH_2_0. Loci GS14, GS17, GS20, and GS22 were amplified with 33 cycles of 94°C for 30 sec, an annealing temperature of 56°C (GS14, GS17), 66°C (GS20), and 64°C (GS22) for 30 sec, then an extension at 72°C for 30 sec, followed by a 30‐min extension at 72°C. Fragment analysis of the PCR products was carried on an Applied Biosystems 3730 genetic analyzer at the Nevada Genomics Center (http://www.ag.unr.edu/genomics/), at the appropriate dilution of PCR product. The genotypes were scored using the program Genemapper v. 3.7., where bins were created using known allele sizes for the microsatellites from the literature with additional bins for novel variation found in the Utah prairie dog.

### Characterization of genetic variation

We used FSTAT (version 2.9.3.2; Goudet 1995) to calculate gene diversity (*H*_e_), number of alleles (A) and allelic richness per locus per colony (*R*_S_), and per locus over all colonies (*R*_T_). Observed heterozygosity (*H*_o_) per locus and over all loci was calculated using Microsatellite toolkit in Excel. Observed and expected levels of heterozygosity were compared between native and transplant populations using two‐tailed Mann–Whitney *U* tests.

### Population genetic structure, relatedness, and effective population size

Recent comparisons among multiple Bayesian clustering techniques suggest that datasets should be analyzed using multiple methods, which together should support a biologically meaningful pattern (Frantz et al. 2009). Contemporary clusters of genotypically similar adults were therefore analyzed using two Bayesian genotype clustering methods (STRUCTURE version 2.3.4 and BAPS version 5.2) (Pritchard et al. 2000; Corander et al. 2008). In STRUCTURE we used an admixture model where individuals with novel genotypes can be identified and assigned to a specific range of potential genotype clusters (*k*) for the six native colonies (1–10) and 1–12 *k* for all colonies combined. We specified a 500,000 burn‐in period followed by five 1,000,000 MCMC replicates per *k* to approximate posterior allelic distributions against which individual genotypes were compared and assigned to a cluster (Pritchard et al. 2000). We used the Δ*k* method of Evanno et al. (2005) to determine the optimal *k*. The Δ*k* method calculates the largest change in the LnP(D) between each pair of *k* and *k*−1 for all tests of *k*. In BAPs, we specified 10,000 input iterations for admixture analysis and ran 10,000 input iterations specifying both 9 and 30 reference individuals per sampling location. We conducted five replicates per *k* for *k* = 1–20.

We used the program FSTAT (version 2.9.3.2; Goudet 1995) to calculate *F*_IS_ within and pairwise *F*_ST_ among colonies and genotype clusters. To test for a pattern of isolation‐by‐distance within and across recovery units, we conducted Mantel tests in GENEPOP 4.2 (Raymond and Rousset 1995; Rousset 2008). AMOVA and PCA were conducted in GenAlEx (6.5; Peakall and Smouse 2006; Peakall and Smouse 2012) in order to characterize the partitioning of genetic variation on the landscape. We also calculated relatedness (*r*) among individuals within each colony using the Lynch and Ritland (1999) method in GenAlEx 6.5.

Effective population size (*N*_e_) was calculated for each colony and genotype cluster using NeEstimator (version 1.3) (Peel et al. 2004). We used the heterozygous excess module based on a single point sample. We chose the heterozygote excess method as our effective number of breeders in any one colony is likely to be small and as such allele frequencies in males and females can by chance (drift) be different producing an excess of heterozygotes in the progeny with respect to Hardy–Weinberg equilibrium expectations (Luikart and Cornuet 1999). We also tested for genetic bottlenecks using the program BOTTLENECK (Cornuet and Luikart 1996) and the single step (SMM) and two‐phase (TPM) mutation models.

## Results

### Sampling

Tissue was collected (June–August 2003 and 2004) from 307 individuals: 33 Adult males, 49 adult females, 116 juvenile males, and 109 juvenile females (see Table 1). The Utah prairie dog is a rodent species with a type 3 survivorship curve. Thus, during the trapping season, post juvenile emergence, most individuals in any Utah prairie dog colony are juveniles.

### Genetic variation

There was no evidence of allelic dropout for any locus in any population/colony. There was evidence for null alleles at two loci, GS20 and EAM35, in one and two colonies respectively, but there were no systematic pattern of null alleles at any locus across all colonies. We compared genetic diversity and allelic richness (i.e., *H*_e_, *R*_T_) for adults only (*N* = 82), juveniles (*N* = 223) and for adults and juveniles (*N* = 307) combined. We found no statistically significant differences in either parameter among groups (*H*_e_, *F* = 0.092, *P* = 0.912, Tukey's test for multiple comparisons *P *≥* *0.909; *R*_T_, *F* = 0.985, *P* = 0.386, Tukey's *P* = 0.454). Allelic richness was low with ≤5 alleles per locus across all colonies and ranged from 2 to 5 alleles per locus for all 10 microsatellite loci and all colonies combined (*R*_S_, *N* = 235, juveniles and adults combined) and from 1 to 3 alleles per locus per colony (with and without the SN colony, our smallest sample size, in the analysis) (Table 3). Average levels of heterozygosity for adults ranged from *H*_o_ = 0.102–0.380, and from *H*_e_ = 0.090–0.329, over all colonies. Juveniles had similar levels of average heterozygosity, *H*_o_ = 0.060–0.272 and *H*_e_ = 0.116–0.304, per locus over all colonies when compared with the adult prairie dogs. The average heterozygosity in the native colonies ranged from *H*_o_ = 0.111–0.357, *H*_e_ = 0.106–0.313 and transplanted colonies *H*_o_ = 0.152–0.229, *H*_e_ = 0.144–0.269 (adults and juveniles combined). Many loci were monomorphic in multiple colonies. The average *H*_e_ and *H*_o_ values over all loci per recovery unit were as follows (adults and juveniles combined); Awapa = 0.283 (SD ± 0.188) and 0.22 (SD ± 0.299), Paunsaugunt = 0.254 (SD ± 0.197) and 0.229 (SD ± 0.329) and West Desert = 0.190 (SD ± 0.168) and 0.167 (SD ± 0.304). Observed and expected heterozygosities did not differ between native and transplant populations (Mann–Whitney *U* = 6.0, 7.0; *P* = 0.201, 0.286 respectively).

**Table 3:** Number of individuals successfully genotyped (*N*), number of alleles observed (A), allelic richness per population and locus total (*R*_S_ and *R*_T_), expected heterozygosity (*H*_E_), observed heterozygosity (*H*_O_), and *F*_IS_ per locus per sampling location for adults, juveniles and both combined. Values with an asterisk are statistically significant *F*_IS_ values (adjusted *P* = 0.00045 based on 2200–2400 randomizations). NA = no analysis

Adults		SN	GB	EC	PF	KV	CCP	DF	WPH1	WPH2	M31	M32	LUND	Total A and *R*_T_
EAM35	*N*	5	5	11	8	8	6	4	6	7	9	0	2	
	A	1	2	2	2	1	2	1	1	2	1		2	2
	*R*_S_	1	1.924	1.904	1.45	1	1.939	1	1	1.505	1		2	1.69
	*H*_E_	0	0.533	0.519	0.233	0	0.545	0	0	0.264	0		0.667	
	*H*_O_	0	0	0	0	0	0	0	0	0	0		0	
	*F*_IS_	NA	1	1*	1	NA	1	NA	NA	1	NA		1	
EAM163	*N*	5	5	9	8	7	6	4	6	4	9	0	2	
	A	1	1	1	1	2	1	1	1	1	1		1	2
	*R*_S_	1	1	1	1	1.286	1	1	1	1	1		1	1.031
	*H*_E_	0	0	0	0	0.143	0	0	0	0	0		0	
	*H*_O_	0	0	0	0	0.143	0	0	0	0	0		0	
	*F*_IS_	NA	NA	NA	NA	0	NA	NA	NA	NA	NA		NA	
GS12	*N*	3	5	11	8	8	6	4	6	7	9	0	14	
	A	2	2	2	2	2	2	2	2	2	2		2	2
	*R*_S_	2	1.952	1.91	1.923	1.912	1.939	1.971	1.939	1.93	1.918		1.898	1.879
	*H*_E_	0.600	0.556	0.524	0.533	0.525	0.545	0.571	0.545	0.538	0.529		0.516	
	*H*_O_	1	1	1	1	0.875	1	1	1	1	1		0.929	
	*F*_IS_	−1	−1	−1	−1	−0.75	−1	−1	−1	−1	‐1		−0.857	
GS14	*N*	5	5	11	8	9	6	4	6	7	9	0	14	
	A	2	2	2	2	1	2	2	2	2	3		2	3
	*R*_S_	1.667	1.924	1.91	1.25	1	1.333	1.971	1.745	1.67	1.627		1.567	1.726
	*H*_E_	0.356	0.533	0.524	0.125	0	0.167	0.571	0.409	0.363	0.307		0.304	
	*H*_O_	0.4	0.4	0.272	0.125	0	0.167	0.500	0.167	0.429	0.333		0.214	
	*F*_IS_	−0.143	0.273	0.492	0	NA	0	0.143	0.615	−0.2	−0.091		0.304	
GS17	*N*	5	5	11	8	8	6	4	6	7	9	0	13	
	A	2	1	1	1	1	1	1	1	1	1		1	2
	*R*_S_	1.924	1	1	1	1	1	1	1	1	1		1	1.095
	*H*_E_	0.533	0	0	0	0	0	0	0	0	0		0	
	*H*_O_	0.800	0	0	0	0	0	0	0	0	0		0	
	*F*_IS_	−0.600	NA	NA	NA	NA	NA	NA	NA	NA	NA		NA	
GS20	*N*	5	5	8	6	8	5	3	4	5	7	0	11	
	A	1	1	2	1	2	1	1	1	1	2		2	2
	*R*_S_	NA	NA	1	NA	1	NA	NA	NA	NA	1		1	
	*H*_E_	0	0	0.4	0	0.233	0	0	0	0	0.263		0.173	
	*H*_O_	0	0	0	0	0	0	0	0	0	0		0	
	*F*_IS_	1	1	1.727	1	1.45	1	1	1	1	1.505		1.338	1.269
GS26	*N*	5	5	11	8	9	6	4	6	7	9	0	14	
	A	3	1	2	2	1	1	1	1	1	1		1	3
	*R*_S_	2.305	1	1.904	1.816	1	1	1	1	1	1		1	1.597
	*H*_E_	0.644	0	0.51948	0.458	0	0	0	0	0	0		0	
	*H*_O_	0.6	0	0.54545	0.375	0	0	0	0	0	0		0	
	*F*_IS_	0.077	NA	−0.053	0.192	NA	NA	NA	NA	NA	NA		NA	
IGS1	*N*	5	4	10	8	6	5	3	6	5	6	0	2	
	A	2	2	2	2	1	1	2	2	3	1		2	4
	*R*_S_	1.400	1.786	1.79	1.45	1	1	2	1.576	2.476	1		2	1.912
	*H*_E_	0.200	0.429	0.442	0.233	0	0	0.600	0.303	0.689	0		0.500	
	*H*_O_	0.200	0	0.400	0.250	0	0	0.333	0.333	0.600	0		0.500	
	*F*_IS_	0	1	0.1	−0.077	NA	NA	0.5	−0.111	0.143	NA		0	
IGS6	*N*	5	5	11	8	7	6	4	6	7	9	0	14	
	A	3	1	1	2	1	1	2	2	2	2		2	4
	*R*_S_	2.229	1	1	1.45	1	1	1.786	1.745	1.67	1.405		1.898	2.18
	*H*_E_	0.600	0	0	0.233	0	0	0.429	0.409	0.363	0.209		0.516	
	*H*_O_	0.800	0	0	0.25	0	0	0	0.500	0.429	0.222		0.500	
	*F*_IS_	−0.391	NA	NA	−0.077	NA	NA	1	−0.250	−0.200	−0.067		0.032	
IGS22	*N*	5	5	10	8	8	6	4	6	7	3	0	12	
	A	2	1	1	1	1	1	1	1	1	1		1	2
	*R*_S_	1.667	1	1	1	1	1	1	1	1	1		1	1.054
	*H*_E_	0.356	0	0	0	0	0	0	0	0	0		0	
	*H*_O_	0	0	0	0	0	0	0	0	0	0		0	
	*F*_IS_	1	NA	NA	NA	NA	NA	NA	NA	NA	NA		NA	
Avg *H*_o_		0.380	0.140	0.222	0.200	0.102	0.117	0.183	0.200	0.246	0.156		0.214	
Avg *H*_*e*_		0.329	0.205	0.293	0.182	0.090	0.126	0.217	0.167	0.222	0.131		0.268	

Juveniles		SN	GB	EC	PF	KV	CC	DF	WP1	WP2	M31	M32	LUND	Total A and *R*_T_
EAM35	*N*	4	11	16	13	21	22	21	20	18	23	26	5	
	A	1	2	2	2	2	2	2	1	2	2	1	2	2
	*R*_S_	1	1.751	1.976	1.415	1.268	1.809	1.474	1	1.695	1.246	1	1.867	1.522
	*H*_E_	0	0.312	0.508	0.093	0.148	0.359	0.177	0.000	0.286	0.085	0	0.356	
	*H*_O_	0	0	0	0	0	0	0	0	0	0	0	0	
	*F*_IS_	NA	1	1*	1	1	1*	1	NA	1	1	NA	1	
EAM163	*N*	4	11	15	14	21	22	21	20	17	24	27	4	
	A	1	1	2	2	1	2	1	1	2	1	1	1	3
	*R*_S_	1	1	1.200	1.530	1	1.136	1	1	1.326	1	1	1	1.103
	*H*_E_	0	0	0.067	0	0.198	0.045	0	0	0.114	0	0	0	
	*H*_O_	0	0	0.067	0	0.071	0.045	0	0	0.118	0	0	0	
	*F*_IS_	NA	NA	0	0.649	NA	0	NA	NA	−0.032	NA	NA	NA	
GS12	*N*	3	13	16	14	23	22	24	20	19	23	27	13	
	A	2	2	2	2	2	2	2	2	2	2	2	2	2
	*R*_S_	2	1.985	1.982	1.982	1.978	1.979	1.978	1.98	1.98	1.978	1.977	1.985	1.97
	*H*_E_	0.600	0.520	0.516	0.511	0.516	0.512	0.511	0.513	0.514	0.511	0.509	0.520	
	*H*_O_	0.600	0.520	0.516	0.511	0.516	0.512	0.511	0.513	0.514	0.511	0.509	0.520	
	*F*_IS_	−1	−1	−1	−0.857	−1	−1	−1	−1	−1	−1	−1	−1	
GS14	*N*	3	13	16	15	23	22	24	20	19	24	26	14	
	A	2	2	2	1	1	2	2	2	2	3	2	2	3
	*R*_S_	2	1.882	1.940	1	1	1.136	1.977	1.394	1.863	1.812	1.540	1.389	1.793
	*H*_E_	0.533	0.409	0.466	0	0	0.045	0.510	0.142	0.398	0.318	0.208	0.138	
	*H*_O_	0	0.385	0.438	0	0	0.045	0.458	0.150	0.211	0.292	0.231	0.143	
	*F*_IS_	1	0.063	0.063	NA	NA	0	0.103	−0.056	0.478	0.085	−0.111	−0.040	
GS17	*N*	3	12	16	11	23	21	23	20	18	24	27	13	
	A	2	1	1	1	1	1	1	1	1	2	1	1	3
	*R*_S_	2	1	1	1	1	1	1	1	1	1.573	1	1	1.111
	*H*_E_	0.533	0	0	0	0	0	0	0	0	0.2234	0	0	
	*H*_O_	0.533	0	0	0	0	0	0	0	0	0.2234	0	0	
	*F*_IS_	−0.333	NA	NA	NA	NA	NA	NA	NA	NA	1*	NA	NA	
GS20	*N*	4	13	10	11	18	19	21	19	15	13	15	14	
	A	1	2	2	2	2	2	2	1	2	2	2	1	2
	*R*_S_	1	1.415	1.793	1.481	1.535	1.513	1.268	1	1.366	1.415	1.773	1	1.401
	*H*_E_	0	0.148	0.337	0.203	0.173	0.193	0.093	0	0.129	0.148	0.331	0	
	*H*_O_	0	0	0	0	0	0	0	0	0	0	0	0	
	*F*_IS_	NA	1	1	1	1	1	1	NA	1	1	1	NA	
GS26	*N*	4	13	16	17	23	22	24	20	19	24	27	13	
	A	2	1	2	3	2	1	2	1	1	2	1	1	4
	*R*_S_	2	1	1.981	1.353	1.903	1	1.125	1	1	1.336	1	1	1.636
	*H*_E_	0.536	0	0.514	0.433	0.116	0	0.042	0	0	0.120	0	0	
	*H*_O_	0.750	0	0.688	0.522	0.118	0	0.042	0	0	0.125	0	0	
	*F*_IS_	−0.5	NA	−0.352	−0.016	−0.211	NA	0	NA	NA	−0.045	NA	NA	
IGS1	*N*	4	11	14	9	23	22	23	20	14	15	13	7	
	A	2	2	2	1	3	1	3	2	3	1	2	3	5
	*R*_S_	1.750	1.481	1.389	1	1.836	1	2.087	1.711	2.537	1	1.764	2.385	1.974
	*H*_E_	0.250	0.173	0.138	0.329	0	0	0.513	0.296	0.593	0	0.323	0.484	
	*H*_O_	0.250	0.182	0.143	0.391	0	0	0.348	0.350	0.429	0	0.385	0.286	
	*F*_IS_	0	−0.053	−0.04	NA	−0.193	NA	0.327	−0.188	0.284	NA	−0.2	0.429	
IGS6	*N*	4	13	16	15	23	22	24	20	19	24	27	14	
	A	2	1	1	2	2	1	3	3	2	2	2	2	4
	*R*_S_	1.75	1	1	1.501	1.752	1	2.083	2.026	1.733	1.237	1.459	1.95	2.241
	*H*_E_	0.250	0	0	0.322	0.186	0	0.513	0.445	0.309	0.082	0.171	0.476	
	*H*_O_	0.250	0	0	0.391	0.067	0	0.083	0.550	0.158	0.083	0.185	0.286	
	*F*_IS_	0	NA	NA	0.65	−0.222	NA	0.841	−0.244	0.495	−0.022	−0.083	0.409	
IGS22	*N*	3	13	16	15	23	22	24	20	19	10	15	14	
	A	2	1	1	1	1	1	1	1	1	2	1	1	3
	*R*_S_	2	1	1	1	1	1	1	1	1	1.793	1	1	1.076
	*H*_E_	0.333	0	0	0	0	0	0	0	0	0.337	0	0	
	*H*_O_	0.333	0	0	0	0	0	0	0	0	0	0	0	
	*F*_IS_	0	NA	NA	NA	NA	NA	NA	NA	NA	1	NA	NA	
Avg *H*_o_		0.272	0.109	0.185	0.182	0.077	0.060	0.144	0.156	0.143	0.123	0.131	0.123	
Avg *H*_e_		0.304	0.156	0.255	0.189	0.134	0.116	0.236	0.140	0.234	0.182	0.154	0.197	

Combined		SN	GB	EC	PF	KV	CC	DF	WPH1	WPH2	M31	M32	LUND	Total A and *R*_T_
EAM35	*N*	9	16	27	29	21	28	25	26	25	30	28	7	
	A	1	2	2	2	2	2	2	1	2	2	1	2	2
	*R*_S_	1	1.988	2	1.615	1.495	1.99	1.679	1	1.998	1.363	1	2	1.863
	*H*_E_	0	0.387	0.503	0.131	0.093	0.416	0.150	0	0.470	0.066	0.000	0.440	
	*H*_O_	0	0	0	0	0	0	0	0	0	0	0	0	
	*F*_IS_	NA	1	1*	1	1	1*	1	NA	1*	1	NA	1	
EAM163	*N*	9	16	24	29	21	28	25	26	21	31	29	6	
	A	1	1	2	1	3	2	1	1	2	1	1	1	3
	*R*_S_	1	1	1.25	1	1.932	1.214	1	1	1.495	1	1	1	1.175
	*H*_E_	0	0	0.042	0	0.180	0.036	0	0	0.093	0	0	0	
	*H*_O_	0	0	0.042	0	0.095	0.036	0	0	0.095	0	0	0	
	*F*_IS_	NA	NA	0	NA	0.477	0	NA	NA	−0.026	NA	NA	NA	
GS12	*N*	6	18	27	31	21	28	28	26	26	30	29	27	
	A	2	2	2	2	2	2	2	2	2	2	2	2	2
	*R*_S_	2	2	2	2	2	2	2	2	2	2	2	2	2
	*H*_E_	0.545	0.514	0.509	0.508	0.507	0.509	0.509	0.510	0.510	0.508	0.509	0.509	
	*H*_O_	1	1	1	1	0.909	1	1	1	1	1	1	0.963	
	*F*_IS_	−1	−1	−1	−1	−0.826	−1	−1	−1	−1	−1	−1	−0.926	
GS14	*N*	8	18	27	31	24	28	28	26	26	31	28	28	
	A	2	2	2	2	1	2	2	2	2	3	2	2	3
	*R*_S_	1.999	1.996	1.999	1.194	1	1.386	2	1.811	1.981	2.257	1.783	1.835	1.999
	*H*_E_	0.400	0.437	0.492	0.032	0	0.070	0.508	0.208	0.382	0.328	0.195	0.223	
	*H*_O_	0.250	0.389	0.370	0.032	0	0.071	0.464	0.154	0.269	0.323	0.214	0.179	
	*F*_IS_	0.390	0.110	0.250	0.000	NA	−0.020	0.090	0.270	0.300	0.020	−0.100	0.200	
GS17	*N*	8.00	17.00	27.00	31.00	19.00	27.00	27.00	26.00	25.00	31.00	29.00	26.00	
	A	2	1	1	1	1	1	1	1	1	2	1	1	3
	*R*_S_	2	1	1	1	1	1	1	1	1	1.742	1	1	1.234
	*H*_E_	0.5	0	0	0	0	0	0	0	0	0.178	0	0	
	*H*_O_	0.75	0	0	0	0	0	0	0	0	0	0	0	
	*F*_IS_	−0.556	NA	NA	NA	NA	NA	NA	NA	NA	1*	NA	NA	
GS20	*N*	9	18	18	25	19	24	24	23	20	18	17	25	
	A	1	2	2	2	2	2	2	1	2	2	2	2	2
	*R*_S_	1	1.562	1.976	1.679	1.797	1.697	1.441	1	1.515	1.82	1.945	1.426	1.633
	*H*_E_	0	0.108	0.356	0.150	0.193	0.156	0.082	0	0.097	0.203	0.299	0.078	
	*H*_O_	0	0	0	0	0	0	0	0	0	0	0	0	
	*F*_IS_	NA	1	1	1	1	1	1	NA	1	1	1	1	
GS26	*N*	9	18	27	31	24	28	28	26	26	31	29	27	
	A	3	1	2	2	3	1	2	1	1	2	1	1	4
	*R*_S_	2.667	1	2	1.995	1.5	1	1.214	1	1	1.482	1	1	1.937
	*H*_E_	0.582	0	0.507	0.444	0.082	0	0.036	0	0	0.094	0	0	
	*H*_O_	0.667	0	0.630	0.516	0.083	0	0.036	0	0	0.097	0	0	
	*F*_IS_	−0.157	NA	−0.249	−0.165	−0.011	NA	0	NA	NA	−0.034	NA	NA	
IGS1	*N*	9	15	24	31	15	27	26	26	19	20	14	9	
	A	2	3	2	3	1	1	3	2	3	1	2	3	5
	*R*_S_	1.902	2.297	1.92	2.098	1	1	2.23	1.926	2.91	1	1.956	2.877	2.734
	*H*_E_	0.209	0.248	0.284	0.302	0	0	0.514	0.292	0.602	0	0.304	0.464	
	*H*_O_	0.222	0.133	0.250	0.355	0	0	0.346	0.346	0.474	0	0.357	0.333	
	*F*_IS_	−0.067	0.472	0.121	−0.179	NA	NA	0.33	−0.19	0.217	NA	−0.182	0.294	
IGS6	*N*	9	18	27	31	20	28	28	26	26	31	29	28	
	A	3	1	1	2	1	1	3	3	2	2	2	2	4
	*R*_S_	2.667	1	1	1.944	1	1	2.213	2.218	1.946	1.482	1.769	2	2.635
	*H*_E_	0.569	0	0	0.317	0	0	0.499	0.429	0.317	0.094	0.189	0.503	
	*H*_O_	0.556	0	0	0.387	0	0	0.071	0.538	0.231	0.097	0.207	0.393	
	*F*_IS_	0.024	NA	NA	−0.224	NA	NA	0.859*	−0.261	0.275	−0.034	−0.098	0.223	
IGS22	*N*	8	18	26	31	23	28	28	26	26	12	16	26	
	A	2	1	1	1	1	1	1	1	1	2	1	1	3
	*R*_S_	1.993	1	1	1	1	1	1	1	1	1.953	1	1	1.153
	*H*_E_	0.325	0	0	0	0	0	0	0	0	0.28986	0	0	
	*H*_O_	0.125	0	0	0	0	0	0	0	0	0	0	0	
	*F*_IS_	0.632	NA	NA	NA	NA	NA	NA	NA	NA	1	NA	NA	
Avg *H*_o_		0.357	0.152	0.229	0.229	0.109	0.111	0.192	0.204	0.207	0.152	0.178	0.187	
Avg *H*_e_		0.313	0.169	0.269	0.189	0.106	0.119	0.230	0.144	0.247	0.176	0.150	0.222	

Ten of the colonies had rare or unique alleles (Table 4). We considered an allele rare if it had a low frequency and/or if it was found in fewer than four colonies. The native colonies had a greater numbers of rare alleles (five colonies, 10 rare alleles) versus transplanted colonies (four colonies, four rare alleles). Although only nine individuals were sampled from the native colony of Smooth Knolls, we found two unique alleles and three rare alleles (Table 4).

**Table 4:** Unique and rare alleles per locus per population, frequencies of allele in colonies in which they are found are listed in parentheses (^1^included due to very low frequency of occurrence in other populations)

Locus		EAM163	GS14	GS17	GS26	IGS1	IGS6	IGS22
Recovery unit	Colony	Unique alleles						
Awapa	SN			160 bp (37.50%)				185 bp (18.75%)
West Desert	KV	89 bp (4.17%)			113 bp (1.16%)			
	M31		247 bp (3.23%)	144 bp (9.68%)				167 bp (16.67%)

		Rare alleles						
Awapa	SN				111 bp (1.6%)	95 bp (*88.89%*)^1^	129 bp (5.56%)	
Paunsaugunt	PF					102 bp (1.61%)		
	EC	80 bp (2.08%)						
West desert	KV	80 bp (1.39%)			111 bp (5.56%)	95 bp (3.45%)		
	CCP	80 bp (1.79%)						
	DF					102 bp (1.92%)		
	WPH1						129 bp (1.92%)	
	WPH2	80 bp (4.76%)						
	LUND					95 bp (3.45%)		

### Relatedness

**Figure 2:**
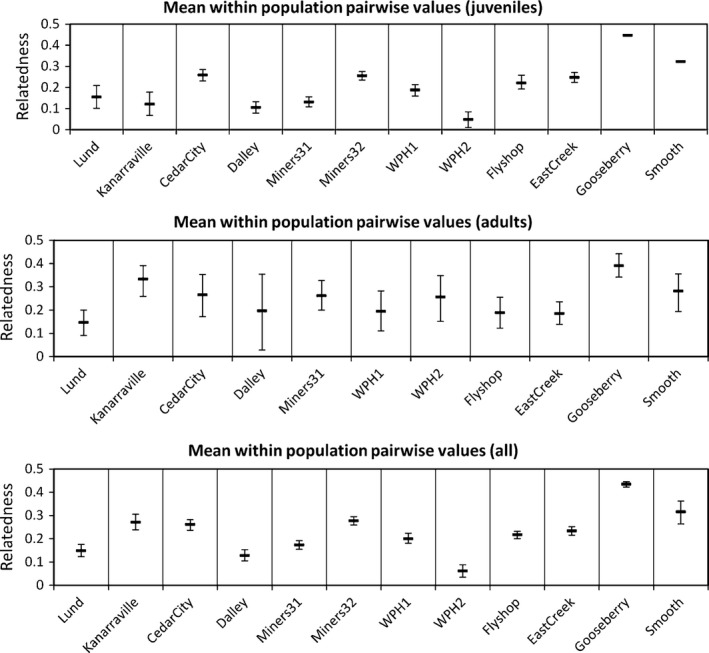
Ritland and Lynch (1999) relatedness (*r*) ± SD per colony for juveniles, adults, and juveniles and adults combined.

Colony relatedness was high with mean *r* values for the native colonies generally higher than the transplanted colonies (Fig. 2). Based upon all pairwise relatedness values, many of the individuals in these colonies could be first order relatives, either full siblings or parent–offspring. *F*_IS_ values for adults within colonies ranged from −1 to 0.492 with four loci monomorphic for the same allele in the majority of colonies (GS17 9/12; GS26 6/12; IGS22 9/12; EAM163 9/10). For juveniles, *F*_IS_ ranged from −1 to 0.495 and three of the four same loci were monomorphic for the same allele in the majority of colonies (GS17 10/12; IGS22 10/12; EAM163 8/12). However, there was only one significant *F*_IS_ value among adults and three among juveniles within colonies (Adults – East Creek, EAM35 *F*_IS_ = 1.0, *P* = 0.0005; Juveniles – Cedar City, East Creek EAM35 *F*_IS_ = 1.0; Dalley Farm IGS6 *F*_IS_ = 0.841; *P* = 0.00042), which suggests random mating among genetically depauperate individuals.

### Population genetic structure

#### Bayesian genotype clustering, PCA, AMOVA

The Bayesian genotype clustering results for adults analyzed separately and for all adults and juveniles combined did not differ so we report on the combined analyses here.

##### Native colonies

**Figure 3:**
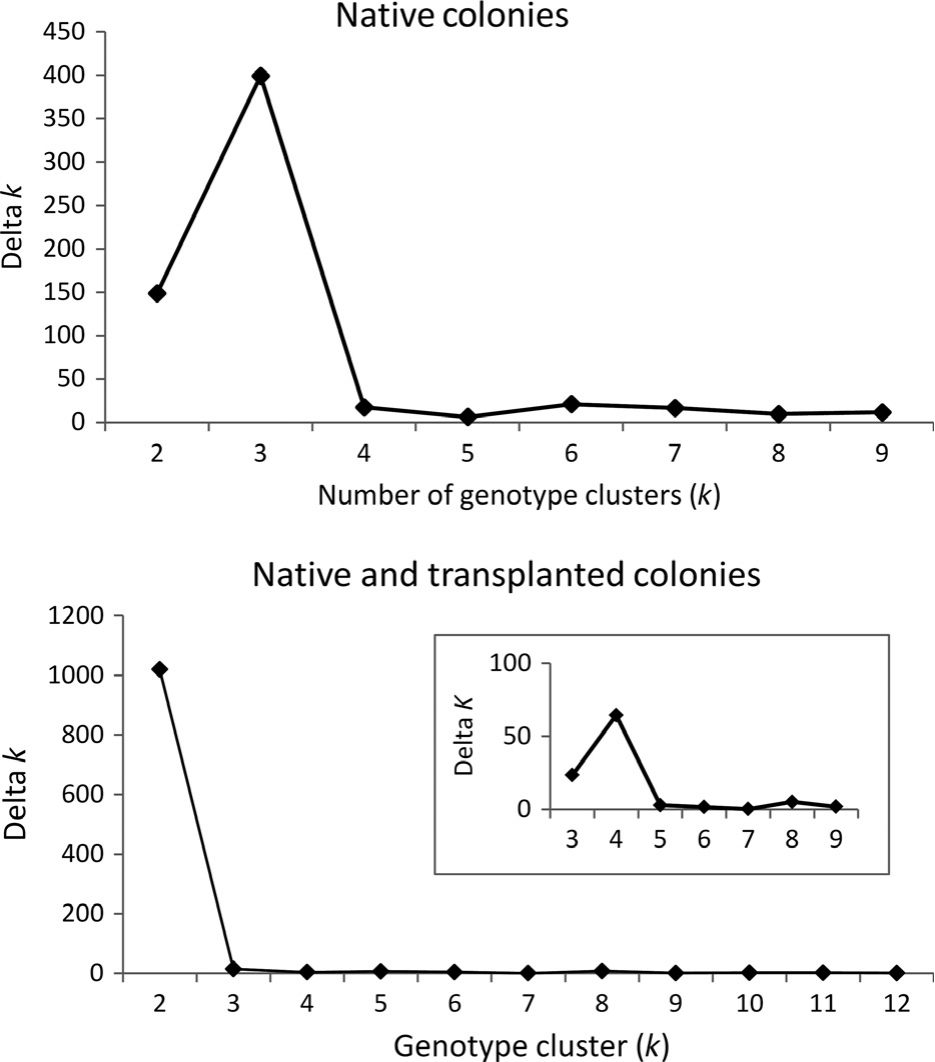
Delta *k* values for Bayesian genotype clustering analysis conducted in STRUCTURE: native colonies and native and transplanted colonies combined.

**Figure 4:**
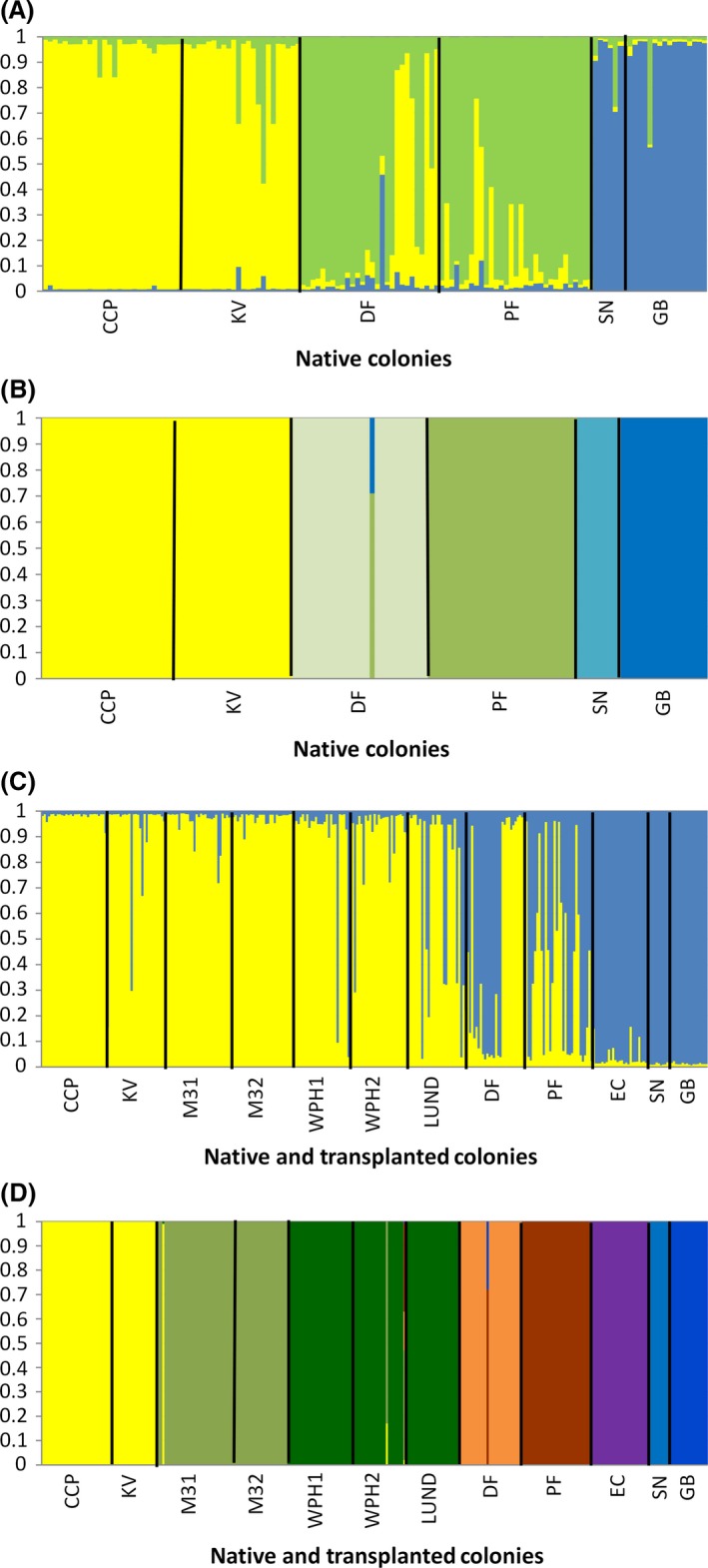
Bayesian genotype clustering output for the best fit of the data: native colonies, (A) STRUCTURE *k* = 3, (B) BAPs *k* = 5; native and transplanted colonies combined (C) STRUCTURE *k* = 2, (D) BAPs *k* = 8.

Three genotype clusters were identified among the native colonies using STRUCTURE [Avg LnP(D) = −876.04, SD = 0.343, Δk = 399.17, *N* = 5 runs per k; Figs 3 and 4]. Individuals from Cedar City and Kanarraville in the West Desert recovery unit formed a single genotype cluster (yellow), Dalley Farm in the West Desert and Panguitch Fly Shop in the Paunsaugunt recovery unit assigned primarily to a second genotype cluster (green) with five individuals from the Dalley Farm colony and two in the Panguitch Fly Shop colony having high assignment to the yellow cluster. The Smooth Knolls and Gooseberry individuals assigned to the third genotype cluster (blue). The BAPs analysis identified five genotype clusters. Cedar City and Kanarraville assigned to the same genotype cluster, but individuals from Dalley Farm, Panguitch Fly Shop, Smooth Knolls, and Gooseberry colonies all assigned to separate clusters. One individual from Dalley Farm had ~70% assignment to the Panguitch Fly Shop genotype cluster and 30% assignment to the Gooseberry cluster.

**Figure 5:**
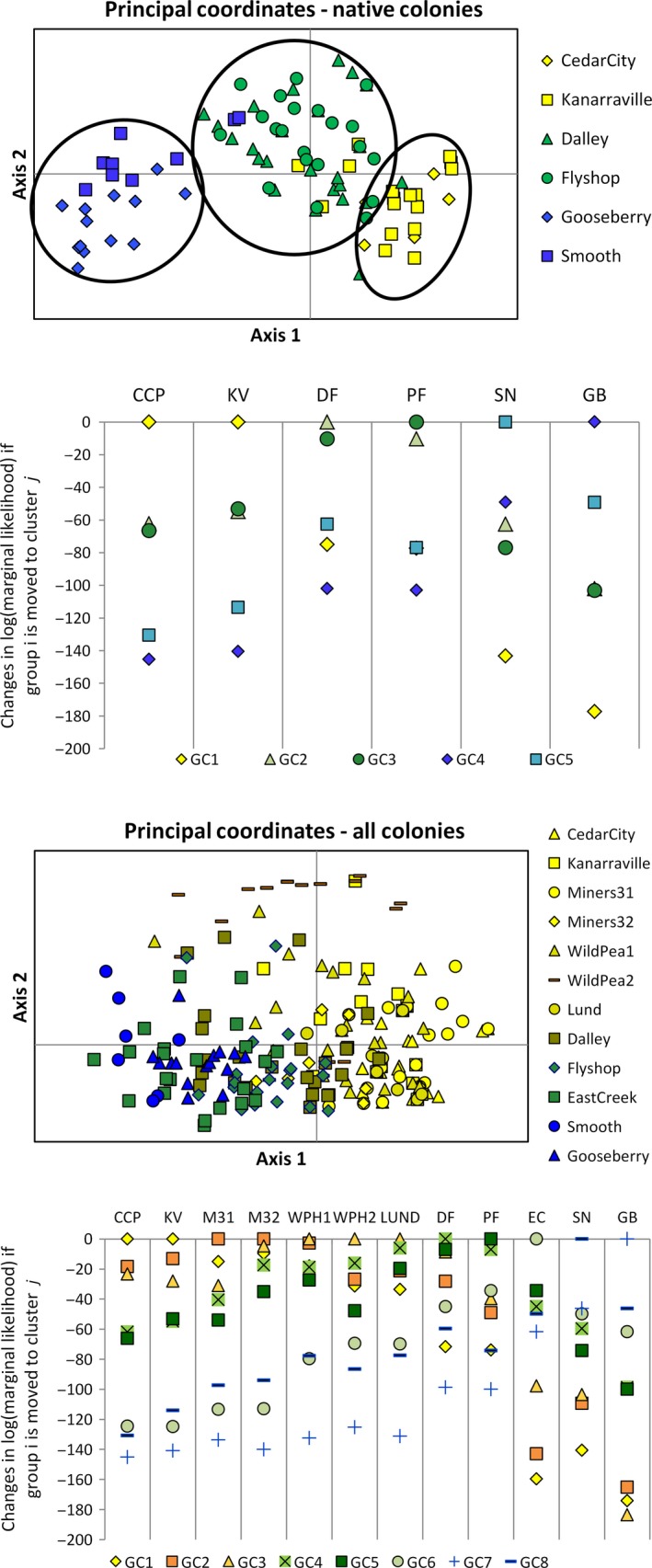
Principle coordinates analysis (PCA) and log(marginal likelihood) if group “i” is moved to group “j” results: native colonies only and all colonies combined.

PCA results show three distinct groups with little overlap: (1) Gooseberry and Smooth Knolls, (2) Dalley Farm and Panguitch Fly Shop, and (3) Cedar City and Kanarraville form (Fig. 5) which supports the STRUCTURE results. Changes in log(marginal likelihood) of assignment if groups are moved to a different cluster show the same pattern (Fig. 5). AMOVA results show that 44% of the molecular variance was within individuals, 22% among individuals, 18% among populations, and 16% among regions.

##### All colonies

Two genotype clusters were identified using STRUCTURE [Avg LnP(D) = −2470.73, SD = 0.485, Δk = 1019.53, *N* = 5 runs per k; Figs 3 and 4]. All of the West Desert colonies (Cedar City, Kanarraville, Minersville31 and 32, WPH1 and 2, and Lund) assigned to a single genotype cluster (yellow) with a few individuals assigning primarily to the second (blue) cluster (Fig. 4). The Minersville31 and 32, and Lund were colonies formed with individuals transplanted from Cedar City and WPH1 and 2 are natural colonizations likely from the Minersville colonies. Approximately half of the Dalley Farm individuals assigned to the yellow and half to the blue cluster. Twelve individuals in the Panguitch Fly Shop colony were well admixed with five individuals assigning to the yellow cluster and thirteen assigning to the blue cluster. All individuals in the East Creek colony in the Paunsaugunt Recovery Unit, and Smooth Knolls and Gooseberry colonies in the Awapa Recovery Unit assigned to the blue cluster, despite the fact that records for the East Creek colony show Cedar City as the source population. There was also some statistical support for *k* = 4 (LnP(D) = −1927.714, Δk = 6.91) in which three genotype clusters are identified for the West Desert Colonies and all individuals were admixed between two of the three genotype clusters. Individuals from the Dalley Farm and Panguitch Fly Shop colonies assign primarily to one of the three clusters present in West Desert, but have some admixture with the genotype cluster that East Creek, Smooth Knolls and Gooseberry assigned to. BAPs identified eight genotype clusters, with Dalley Farm, Panguitch Fly Shop, East Creek, Smooth Knolls, and Gooseberry assigning to distinct clusters. Unlike with the native colonies there was substantial overlap in PCA space among the colonies in the Pausaugunt and Awapa Recovery Units. However, changes in log(marginal likelihood) of assignment if groups are moved to a different cluster show a clearer pattern of separation among the colonies (Fig. 5). AMOVA results were similar to those for the native colony analysis with 41% of the molecular variance within individuals, 23% among individuals, 17% among populations, and 19% among regions.

### *F* statistics

Although pairwise *F*_ST_ values were quite high, there were no significant pairwise *F*_ST_'s among colonies when adults were analyzed separately, but sample sizes were quite small (see Table 1). However, there were significant pairwise *F*_ST_'s among colonies for juveniles analyzed alone and for all individuals combined (Table 5). Nm estimates based upon *F*_ST_ values suggest very few dispersers among colonies (Table 6). Smooth Knolls, Gooseberry, East Creek, Panguitch Fly Shop, and Dalley Farm colonies were significantly differentiated from most of the other colonies. *F*_ST_ values were much lower and nonsignificant among colonies within the West Desert Recovery Unit. Global *F*_ST_ [Weir and Cockerham's (1984) *θ* calculated in FSTAT] was 0.318 for adults, 0.279 for juveniles and 0.296 for adults and juveniles combined. All pairwise *F*_ST_ estimates were significant among genotype clusters determined by STRUCTURE and BAPs for native colonies (STRUCTURE *P *=* *0.017, 60 permutations; BAPS *P *=* *0.005, 200 permutations) and for native and transplanted colonies combined (STRUCTURE *P *=* *0.008, 120 permutations; BAPs *P *=* *0.001, 560 permutations). All recovery units were also significantly differentiated from each other (Awapa‐Paunsaugunt *F*_ST_ = 0.326; Awapa‐West *F*_ST_ = 0.433; Paunsaugunt‐West *F*_ST_ = 0.21; adjusted *P *=* *0.016, obtained after 60 permutations).

**Table 5:** Pairwise *F*_ST_ values that are bolded, and with an asterisk are statistically significant and represent genetically differentiated colonies (adjusted *P* = 0.0007 obtained after 1320 permutations; adults and juveniles combined)

	GB	SN	EC	PF	KV	CCP	DF	WPH1	WPH2	M31	M32	Lund
GB												
SN	0.4132											
EC	0.3529*	0.3139*										
PF	0.4941*	0.4265	0.1994*									
KV	0.6474	0.6105	0.4751	0.325								
CCP	0.6365*	0.6146*	0.4599*	0.3407*	0.0359							
DF	0.4666*	0.3184*	0.2109*	0.1518*	0.2947	0.302*						
WPH1	0.5877*	0.5066*	0.3779*	0.1977*	0.0881	0.1231*	0.1579*					
WPH2	0.4925*	0.4*	0.2986*	0.2373*	0.1445	0.104*	0.1216*	0.0861				
M31	0.5662	0.5096	0.4109	0.2855	0.0335	0.0634	0.228	0.0681	0.1229			
M32	0.6002	0.5366	0.4202	0.2612	0.0341	0.0814	0.188	0.0477	0.0948	0.0286		
Lund	0.5058*	0.4183	0.2525*	0.1193	0.1529	0.116	0.0736	0.0657*	0.0217	0.1409	0.0992	

**Table 6:** Pairwise Population Nm Values Based on *F*_ST_

	Lund	KV	CCP	M31	M32	WPH1	WPH2	Dalley	Flyshop	EC	GB	SN
Lund	0.000											
Kanarraville	0.740	0.000										
CedarCity	0.346	2.814	0.000									
Miners31	0.412	1.623	0.900	0.000								
Miners32	0.478	2.346	0.880	16.378	0.000							
WildPea1	0.366	2.321	2.332	0.900	0.981	0.000						
WildPea2	0.942	4.524	2.077	1.247	1.788	2.056	0.000					
Dalley	0.620	1.208	0.781	0.750	0.896	1.608	2.509	0.000				
Flyshop	0.427	0.891	0.630	0.553	0.601	1.219	0.978	1.832	0.000			
EastCreek	0.411	0.484	0.364	0.424	0.428	0.482	0.762	1.255	1.274	0.000		
Gooseberry	0.236	0.290	0.189	0.249	0.234	0.217	0.350	0.391	0.328	0.554	0.000	
Smooth	0.297	0.378	0.196	0.339	0.334	0.274	0.475	0.651	0.395	0.615	0.468	0.000

### Mantel

**Figure 6:**
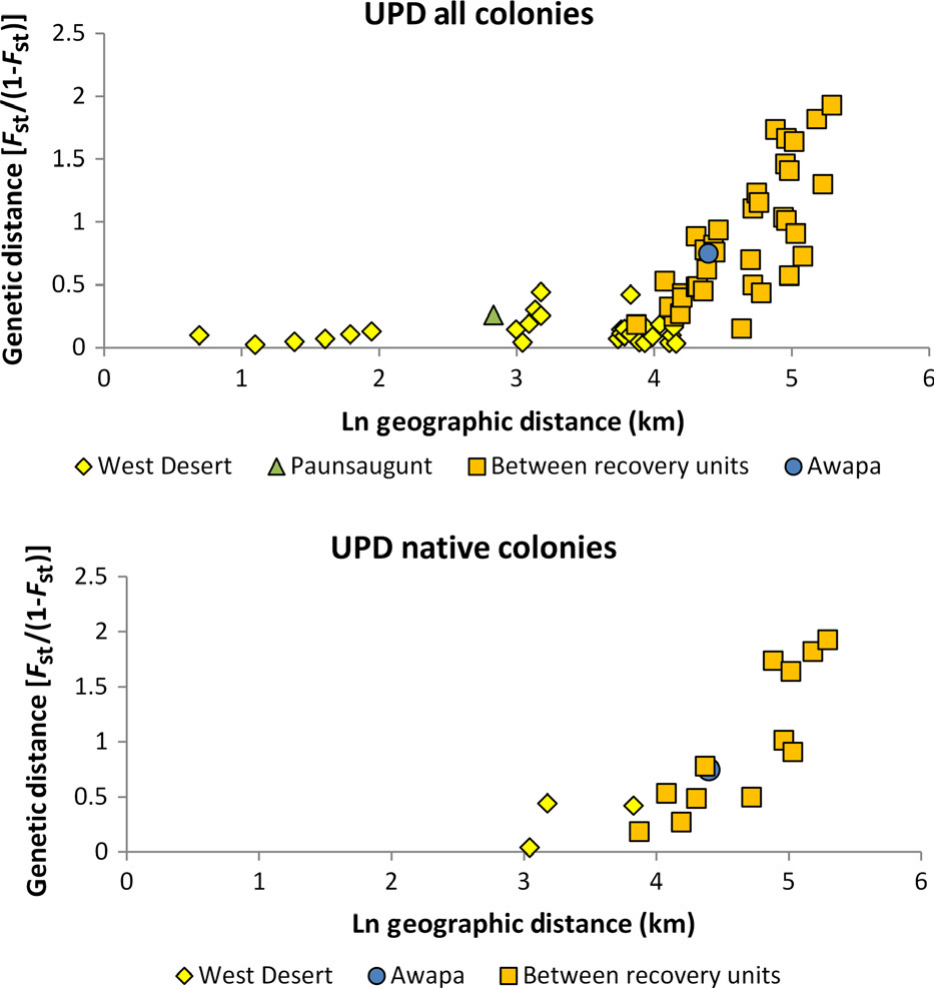
Mantel test comparing genetic and geographic distance: all pairwise colony comparisons and native colony pairwise comparisons only.

We did not observe an isolation‐by‐distance pattern for all pairwise colony comparisons within and among recovery areas (*F* = 1.701, *P *=* *0.203; Fig. 6). This was not surprising given that most of the West Desert colonies sampled were found with individuals from the Cedar City colony. However, pairwise comparisons among native colonies for within and among recovery units were highly significant (*F* = 45.358, *P *=* *0.000; Fig. 6).

### Genetic bottlenecks and effective population size

All populations showed evidence of genetic bottlenecks under both the SMM and TPM mutation models (*P *≤* *0.018, range 0.002–0.018). Effective population sizes were exceedingly small for all colonies sampled ranging from 1.9 (PF) to 13.9 (SN) per colony (GB = 2.9, EC = 4.1, KV = 2.2, CCP = 2.3, DF = 3.7, WP1 = 2.0, WP2 = 3.6 M31 = 4.3, M32 = 7.0, and LUND = 3.2), suggesting small founder populations and/or extensive losses of genetic variation through random genetic drift when populations were small. *N*_e_ was also small for the genotype clusters identified among the native colonies by STRUCTURE (GB and SN = 15.2, DF and PF = 3.7, and CCP and KV = 2.5).

## Discussion

Prairie dog species have undergone significant anthropogenic‐mediated declines over the 20th and 21st centuries (Collier and Spillett 1973; Scott‐Morales et al. 2005; Sackett et al. 2012; Castellanos‐Morales et al. 2014). In 1973, the Utah prairie dog was listed as endangered under the United States Endangered Species Act. Once the large number of prairie dogs found on unprotected private agricultural land was accounted for, the Utah prairie dog was down listed to ESA threatened status in 1984.

However, despite demographic increases, the results of this study show that genetic effective population sizes are significantly lower than what has been reported for black‐tailed prairie dog colonies, the only other published estimate for a prairie dog species from genetic data (*N*_e_ = 88; Dobson et al. 2004). Levels of allelic diversity and observed heterozygosity for the 10 microsatellite loci used in this study were very low (average per locus per colony: A = 1–3 alleles, *H*_o_ = 0.13–0.36). Overall, the allelic diversity at these microsatellite markers in Utah prairie dog was lower than the species the microsatellites were developed from (GS12‐26 loci 3–11, IGS1&6 loci 3, and EAM35 and 163 loci 5, 9 alleles) but not substantially so. In contrast, the black‐tailed prairie dog has maintained genetic variation despite extensive habitat fragmentation (Lomolino et al. 2003; Antolin et al. 2006; Magle et al. 2010; Castellanos‐Morales et al. 2014).

The Utah prairie dog together with the Mexican prairie dog remains listed as endangered on the IUCN Red List. Comparison of levels of genetic variation found in the Utah prairie dog with the data available for other ground‐dwelling sciurids, which have IUCN vulnerable or endangered status, including the European ground squirrel (vulnerable, *Spermophilus citellus*), Idaho ground squirrel (endangered, *Urocitellus brunneus*), Mohave ground squirrel (vulnerable, *Xerospermophilus mohavensis*), and Perote ground squirrel (endangered, *Xerospermophilus perotensis*), reveals that the Utah prairie dog is among the most genetically depauperate (Bell and Matocq 2011; Hoisington‐Lopez et al. 2012; Ochoa et al. 2012; Ćosić et al. 2013). Ćosić et al. (2013) genotyped 157 European ground squirrels from nine locations at 12 microsatellite loci and allelic diversity ranged from 2 to 18 alleles per locus with expected heterozygosities ranging from 0.406 to 0.581 per population. The Perote ground squirrel is found in only 16 locations reduced from an original range of 5250 km^2^ (Ochoa et al. 2012). Despite the significantly reduced range for the Perote ground squirrel, allelic diversity for six microsatellite loci has remained high and comparable with historical samples (current 5–13 alleles per locus, 36 alleles total, average expected heterozygosity 0.686–0.735; historical 35 alleles total, average expected heterozygosity 0.709; Ochoa et al. 2012). Although Hoisington‐Lopez et al. (2012) do not report number of alleles per microsatellite locus for Idaho ground squirrels sampled from 14 locations (*N* = 339), allelic richness ranged from 2.1 to 3.51 (based upon a sample size of six) and expected heterozygosity per population ranged from 0.393 to 0.658. Despite the vulnerable or endangered status of these species, they are harboring considerably more genetic variation than the Utah prairie dog.

Results of genetic analysis reported here reflect the huge population declines that occurred over the past century, with concomitant losses of genetic variation in Utah prairie dog colonies. This is also reflected in the high relatedness values for individuals within colonies, which because we had very few departures from Hardy–Weinberg equilibrium, suggest random mating among genetically depauperate individuals. Genetic estimates of effective population size are measurements of an “ideal” population under Hardy–Weinberg equilibrium (no migration, mutation, assortative mating, or natural selection) that loses genetic variation due to random genetic drift at the same rate as observed in the real population (Wright 1938, 1940). The extremely low genetic effective population sizes and evidence of genetic bottlenecks strongly suggest that demographic increases occurred from very small remnant populations, where standing genetic variation post genetic bottleneck was likely further eroded by random genetic drift while populations remained small.

The ESU concept has been widely embraced by the conservation biology community (Moritz 1994; Peacock et al. 2010; Bristol et al. 2013; Lumley and Cusson 2013; Olivares et al. 2013; Stockwell et al. 2013). Evolutionarily divergent populations, even if divergence is determined with noncoding genetic markers, likely encompass adaptive differences (Fraser and Bernatchez 2001; Hedrick et al. 2001). However, because ESUs are hard to define in practice, state and federal management agencies use a distinct population segment (DPS) framework to develop management plans for threatened and endangered species with the aim to maintain distinct lineages and adaptive potential. The three recovery units for Utah prairie dog were identified as geographically distinct areas separated by potential barriers to movement with landscape level (elevation and environmental) differences.

Our Bayesian analysis of the native colonies support 3–5 genotype clusters depending upon the clustering algorithm used (STRUCTURE vs. BAPs; Fig. 4). The STRUCTURE results support three genotype clusters, but the spatial extent of these clusters does not overlap with the recovery unit designation of the colonies in all cases. Individuals from Dalley Farm, which is in the West Desert Recovery Unit and the Panguitch Fly Shop in the Paunsaugunt Recovery Unit, assigned to the same genotype cluster, while the remaining native colony assignments were consistent with their recovery unit designation. The BAPs analysis suggests little admixture and five distinct clusters. However, other studies have shown that BAPs has a tendency to overestimate genetic structure (Latch et al. 2006; Rowe and Beebee 2007; Frantz et al. 2009). PCA is consistent with the STRUCTURE results, which provides additional support for three genotype clusters. Despite the overestimation of genetic structure, the BAPs analysis also does not assign Dalley Farm individuals to the same genotype cluster as the other West Desert Recovery Unit colonies. Both Bayesian analyses suggest historical substructure within the West Desert Recovery Unit and gene flow among the easternmost colonies in West Desert with colonies in the western Paunsaugunt Recovery Unit. Historical gene flow is further supported by the presence of abundant suitable habitat for prairie dogs found across the eastern and western portions of the West Desert and Paunsaugunt Recovery Units respectively (see Fig. 1). Contemporary gene flow between Panguitch Valley and Parowan Valley, where Dalley Farm is located, is possible as they are connected by two intermediary valleys (Bear Valley and Bucksin Valley, the type locality for the species (Allen 1905), that are currently occupied by Utah prairie dogs. Although mean dispersal distance measured by mark–recapture methods was only 0.56 km (range 0.16–1.2 km; Mackley et al. 1988), dispersal distances of up to 16 kilometers have been observed (Brown et al. 2011, Utah Division of Wildlife Resources, pers. comm. 2014), suggesting that occasional long‐distance dispersal events do occur. The Panguitch Fly Shop colony is located in the Sevier River Valley which is comprised of long continuous prairie dog habitat, whereas East Creek is located at a higher elevation on the Paunsaugunt plateau. These landscape features and our genetic results suggest that gene flow between these colonies is likely to be rare despite being grouped into the same recovery unit.

When all colonies, native and transplanted, are combined the STRUCTURE results provide strong support for two distinct genetic clusters separating the West Desert colonies with the exception of Dalley Farm, from the remaining colonies which assign primarily to the second genotype cluster. Individual assignment is split between the two genotype clusters for individuals in the Dalley Farm and Panguitch Fly Shop colonies with multiple admixed individuals, whereas East Creek, Smooth Knolls, and Gooseberry assign to the second genotype cluster with little to no admixture present. There was also some statistical support for *k* = 4 which show similar proportional assignment of Dalley Farm and Panguitch Fly Shop individuals to the same clusters.

Interestingly, records for East Creek in the Pausaugunt Recovery Unit identify a Cedar City origin for this colony which is not supported by the data. This result suggests that the Cedar City prairie dogs did not survive in the East Creek site, which was subsequently colonized by prairie dogs from nearby colonies. The majority of transplanted colonies included in the analysis are found in the West Desert Recovery Unit and their cluster assignment reflects their Cedar City origins. Similar to the analysis of native colonies only, additional genotype clusters were identified by BAPs. However, PCA results are consistent with the STRUCTURE results.

The pairwise *F*_ST_ analysis shows highly significant levels of genetic differentiation among colonies both within and among the recovery units. Although *F*_ST_ values using different microsatellite loci are not directly comparable among studies, the pairwise *F*_ST_ values we report here are much higher among Utah prairie dog colonies than for other prairie dog species such as black‐tailed prairie dog where Roach et al. (2001) reported a global *F*_ST_ of 0.118 among thirteen colonies in Colorado using data for seven microsatellite loci some of which were used in this study (but see Magle et al. 2010). Sackett et al. (2012) report pairwise *F*_ST_ estimates ranging from 0.054 to 0.133 among 10 colonies of black‐tailed prairie dog using 11 microsatellites. These estimates are much lower than the global *F*_ST_ (*θ* = 0.296) we report here or Meirmans and Hedrick's (2011) corrected G”_ST_ (= 0.383 for all colonies combined) for Utah prairie dogs. The high and significant *F*_ST_ values we observed may be the result of historical genetic structure, genetic bottlenecks, and the recent effects of random genetic drift at small population sizes. The pattern of isolation‐by‐distance among recovery units further suggests low rates of contemporary gene flow between these areas.

The presence of unique and rare alleles also supports genetic differentiation and colony isolation. The Smooth Knolls colony, which is quite isolated from the other colonies sampled, has two unique alleles and the highest number of rare alleles despite a very small census size. The transplanted populations maintain similar levels of heterozygosity compared to the native colonies and also contain unique and rare alleles. Together these data paint a picture of three genetically distinct groups comprised of colonies that are now largely isolated.

## Conclusions and Conservation Implications

Humans have decimated Utah prairie dog populations through direct elimination campaigns and habitat destruction/fragmentation/conversion. The remaining colonies have extremely low levels of genetic variation and very small effective population sizes. The human population in southern Utah is growing and the Utah prairie dog habitat will continue to face increasing development pressure which could lead to additional losses of genetic diversity. For a species that is already genetically compromised, this would be disastrous.

Wal et al. (2013) recently suggested that evolutionary rescue – improving adaptive potential – will not occur without intervention for most threatened and endangered vertebrate species due to low population size, long generation times, and limited genetic variability. Genetic rescue of declining lineages is not a new concept, and managed dispersal (gene flow) programs have been implemented in many threatened and endangered species (e.g., Florida Panther; Pimm et al. 2006). Evolutionary rescue of genetically depauperate Western European lineages of Eurasian beaver may require mixing individuals from Eastern ESUs with the Western Europe ESUs (Halley 2011). However, Wal et al. (2013) further suggest that evolutionary rescue should be studied by mapping genotype, phenotype, demography, and fitness relationships, and use this information to set priorities for applying evolutionary rescue to wild populations.

Due to the low overall genetic diversity within the Utah prairie dog species as a whole, human‐mediated translocations among recovery units is not likely to increase diversity. At a minimum there are three distinct genotype clusters among the native colonies representing the repository of extant variation of the species. Our analyses suggest three management actions that might improve gene flow and maximize maintenance of remaining genetic diversity in Utah prairie dogs within and among the recovery units: (1) the protection and maintenance of suitable habitat between existing colonies which could provide critical corridors for dispersal and subsequent gene flow. The small population sizes of most of the colonies sampled for this study suggests that maintenance of unique and rare alleles as well as overall genetic diversity can only be achieved through increases in population size and dispersal among neighboring populations within recovery units. We emphasize that populations in the Pausaugunt recovery unit, although biogeographically distinct, are not a genetic entity. Landscape genetic analyses of the black‐tailed prairie dog show that introduction of plague and habitat fragmentation over the 20th century has resulted in semi‐isolated populations that now function as metapopulations (Antolin et al. 2006; Magle et al. 2010). Dispersal among black‐tailed prairie dog towns is facilitated by low lying drainages which function as dispersal corridors (Antolin et al. 2006). The ability to disperse among colonies has resulted in maintenance of considerable genetic variation for this species. Bell and Matocq (2011) document historical gene flow and connectivity among populations of Mohave ground squirrels and suggest that current management goals should be to identify habitat corridors that promote population connectivity to the greatest extent possible. (2) Increased attention should be paid to the placement of translocation populations with a focus on proximity to existing prairie dog colonies and suitable but unoccupied habitat. The Wild Pea Hollow populations are thought to be the results of a natural colonization from geographically proximate colonies. This result suggests that prairie dog colonies may act as metapopulations, similar to what has been observed in the black‐tailed prairie dog (Antolin et al. 2006; Magle et al. 2010), whereby locally extirpated colonies can be naturally reestablished; and (3) the construction of translocation complexes which would facilitate local gene flow. Large translocation sites comprised of multiple independent release sites should be located within average dispersal distances of one another. The genetic results presented here suggest that prairie dogs from Dalley Farm and Panguitch Fly Shop should be used to form new colonies in the Sevier River Valley, whereas Prairie dogs from the East Creek, Smooth Knolls, and Gooseberry colonies should be used to found additional colonies in close proximity within their respective Recovery Units.

In the short term, genetic monitoring of prairie dog colonies should be undertaken to assess ongoing maintenance of genetic variation at neutral microsatellite markers and guide targeting of juvenile animals for translocation in order to increase frequencies of unique and rare alleles and maximize population levels of heterozygosity. The high rates of predation and the presence of cannibalism may require multiple translocation efforts of juvenile prairie dogs each year. The Utah prairie dog sits in a precarious position though overall prairie dog numbers appear to be increasing this is largely a result of increases in private land in the West Desert Recovery area. The Awapa and Paunsaugunt recovery units have currently only small numbers of prairie dogs (<1000 individuals in the Awapa, <2000 in the Paunsaugunt), but the prairie dog populations appear to be stable (adult spring count; USFWS, 2012; Utah Division of Wildlife Resources, unpubl. data, 2014). Increases in the number of and/or gene flow among prairie dog colonies especially within Awapa and Paunsaugunt recovery units would help to secure the unique genetic variation found in these colonies.

## Conflict of Interest

None declared.
